# Children and Adults in a Household Cohort Study Have Robust Longitudinal Immune Responses Following SARS-CoV-2 Infection or Exposure

**DOI:** 10.3389/fimmu.2021.741639

**Published:** 2021-10-13

**Authors:** Melanie R. Neeland, Samantha Bannister, Vanessa Clifford, Jill Nguyen, Kate Dohle, Isabella Overmars, Zheng Quan Toh, Jeremy Anderson, Celeste M. Donato, Sohinee Sarkar, Lien Anh Ha Do, Conor McCafferty, Paul V. Licciardi, Vera Ignjatovic, Paul Monagle, Julie E. Bines, Kim Mulholland, Nigel Curtis, Sarah McNab, Andrew C. Steer, David P. Burgner, Richard Saffery, Shidan Tosif, Nigel W. Crawford

**Affiliations:** ^1^ Infection and Immunity Theme, Murdoch Children’s Research Institute, Parkville, VIC, Australia; ^2^ Department of Paediatrics, The University of Melbourne, Parkville, VIC, Australia; ^3^ Infectious Diseases Unit, The Royal Children’s Hospital, Parkville, VIC, Australia; ^4^ Laboratory Services, The Royal Children’s Hospital, Parkville, VIC, Australia; ^5^ Clinical Sciences Theme, Murdoch Children’s Research Institute, Parkville, VIC, Australia; ^6^ Clinical Haematology, The Royal Children’s Hospital, Parkville, VIC, Australia; ^7^ Kids Cancer Centre, Sydney Children’s Hospital, Randwick, NSW, Australia; ^8^ Department of Gastroenterology and Clinical Nutrition, Royal Children's Hospital, Parkville, VIC, Australia; ^9^ Infectious Diseases Epidemiology Department, London School of Hygiene and Tropical Medicine, London, United Kingdom; ^10^ Department of General Medicine, The Royal Children’s Hospital, Parkville, VIC, Australia

**Keywords:** COVID - 19, pediatrics, non-COVID-19 respiratory virus, household contact, cell profile

## Abstract

Children have reduced severity of COVID-19 compared to adults and typically have mild or asymptomatic disease. The immunological mechanisms underlying these age-related differences in clinical outcomes remain unexplained. Here, we quantify 23 immune cell populations in 141 samples from children and adults with mild COVID-19 and their PCR-negative close household contacts at acute and convalescent time points. Children with COVID-19 displayed marked reductions in myeloid cells during infection, most prominent in children under the age of five. Recovery from infection in both children and adults was characterised by the generation of CD8 T_CM_ and CD4 T_CM_ up to 9 weeks post infection. SARS-CoV-2-exposed close contacts also had immunological changes over time despite no evidence of confirmed SARS-CoV-2 infection on PCR testing. This included an increase in low-density neutrophils during convalescence in both exposed children and adults, as well as increases in CD8 T_CM_ and CD4 T_CM_ in exposed adults. In comparison to children with other common respiratory viral infections, those with COVID-19 had a greater change in innate and T cell-mediated immune responses over time. These findings provide new mechanistic insights into the immune response during and after recovery from COVID-19 in both children and adults.

## Introduction

Children have reduced severity of COVID-19 compared to adults, with mild or asymptomatic infection common in this age group (1, 2). Children also have lower transmission rates compared to adults, in part because asymptomatic COVID-19 is less infectious than symptomatic disease (3), and because they are less likely to be the primary index case in a household (4–6). This is in contrast to the higher prevalence and severity observed in children for most other respiratory viruses (1, 7, 8).

Understanding the immunological basis for these age-related differences (9–11), as well as the factors that contribute to protection in household contacts who remain SARS-CoV-2 PCR negative despite close and often prolonged exposure (12), would help accelerate the development of diagnostic and therapeutic strategies to control COVID-19.

In this study, we used high parameter flow cytometry to comprehensively delineate the longitudinal circulating immune cell profiles of children and adults with mild COVID-19 and their close household contacts who were repeatedly PCR negative. To investigate differences in immune responses to SARS-CoV-2 and other respiratory viruses in children, we also compared the immune response in children with SARS-CoV-2 infection alone, SARS-CoV-2 co-infection, and non-SARS-CoV-2 respiratory viral infections.

## Materials and Methods

### Study Cohort

Participants were families presenting for SARS-CoV-2 testing at the Royal Children’s Hospital Melbourne Australia, between April and September 2020 (**Table 1**). A total of 141 samples from SARS-CoV-2-positive or SARS-CoV-2-exposed but PCR negative children and adults were included in the study (complete cohort). Acute samples were collected within 2 weeks of the first SARS-CoV-2 PCR test result and follow up samples were collected 4 to 9 weeks after first SARS-CoV-2 PCR test result. SARS-CoV-2-positive individuals were non-hospitalised patients who had a positive PCR test for SARS-CoV-2 on nasopharyngeal swab and were asymptomatic or had mild symptoms, including coryza, headaches, nausea, fever, cough, sore throat, malaise, and muscle aches (**Table 1**). SARS-CoV-2-exposed individuals were SARS-CoV-2 PCR negative on repeated nasopharyngeal swabs and were close contacts of confirmed SARS-CoV-2-positive patients in their households. Close contact was defined as face-to-face contact for more than 15 minutes and shared closed space with a confirmed case of COVID-19, in accordance with Victorian state guidelines. All participants in the exposed group had up to five repeat SARS-CoV-2 PCR tests at 5-to-7-day intervals for 4 weeks, all of which were negative. SARS-CoV-2 positive children with samples collected during the acute phase were younger than SARS-CoV-2 exposed children (median age 2years *vs* 9years, p=0.02), however no other age or sex differences were found between groups (see Extended Data). SARS-CoV-2 positive children and adults were more likely to be symptomatic relative to SARS-CoV-2 exposed children and adults (**Table 1**, Extended Data). In a subset of 41 participants, paired acute and follow up samples were available for longitudinal analysis (**Table 1**).

**Table 1:** Demographics of study cohort.

	CHILDREN				ADULTS			
	SARS-CoV-2-positive		SARS-CoV-2-exposed		SARS-CoV-2-positive		SARS-CoV-2-exposed	
	Acute	Follow up	Acute	Follow up	Acute	Follow up	Acute	Follow up
**Complete Cohort**								
Number	12	20	7	16	15	15	24	32
Age (years), median (min-max)	2 (1–14)	3.5 (1–17)	9 (3–17)	6 (1–17)	35 (19–62)	36 (20–62)	38 (21–56)	40.5 (22–57)
Sex (% Male)	4 (33.3)	10 (50)	2 (28.5)	5 (31.2)	8 (53.3)	6 (40)	10 (41.6)	16 (50)
Time of sampling since first SARS-CoV-2 PCR result (days), median (min-max)	9 (3–13)	38.5 (31–65)	0 (0–12)	37 (27–44)	11 (4–18)	41.5 (26–52)	4.5 (0–15)	32 (29–65)
% Symptomatic* (n)	91.6 (11)	85 (17)	28.5 (2)	37.5 (6)	86.6 (13)	93.3 (14)	37.5 (9)	53.1 (17)
Number of participants with respiratory panel testing (n)	12	15	7	13	12	13	23	23
Number positive for other respiratory virus (n)	4	8	4	7	0	2	9	11
Time of sampling since respiratory panel result (days), median (min-max)	2.5 (0–8)	32 (23–57)	7 (0–7)	30 (16–37)	5.5 (1–8)	33 (24–44)	2 (0–11)	31 (23–57)
**Longitudinal Sub-Cohort**								
Number	8		7		10		16	
Age (years), median (min-max)	2.5 (1–14)		9 (3–17)		38 (27–62)		38.5 (25–52)	
Sex (% Male)	2 (25)		2 (28.5)		5 (50)		6 (37.5)	
Time of sampling since first PCR test (days), median (min-max)	8.5 (3–13)	38.5 (30–65)	0 (0–12)	27 (27–44)	12 (4–18)	41.5 (26–51)	5 (0–15)	34.5 (27–65)
% Symptomatic* (n)	87.5 (7)		28.5 (2)		90 (9)		50 (8)	

*Symptomatic definition: 2 or more symptoms, 2 days before or within 14 days after swab. Symptoms include coryza, headaches, nausea, fever, cough, sore throat, malaise, and muscle aches.

Additional testing for respiratory pathogens using a commercial multiplex assay (including influenza A/B, respiratory syncytial virus (RSV) A/B, rhinovirus, enterovirus, parechovirus, adenovirus, human parainfluenza viruses 1-4, human metapneumovirus, *Bordetella pertussis* and *Mycoplasma pneumoniae*) was also available for most participants (AusDiagnostics Respiratory Pathogens 16-well assay). Of these pathogens, some participants had evidence of RSV A/B, rhinovirus, enterovirus or adenovirus infection (see extended data for individual results) whilst all other pathogens were not detected in any sample. **Table 1** describes the number of participants with available respiratory panel results, the number of participants in each group positive for other respiratory viruses (other than SARS-CoV-2), and the timing of sample collection relative to respiratory panel testing.

### Diagnosis of SARS-CoV-2 Infection and Other Respiratory Pathogens

Combined oropharyngeal and nasopharyngeal (or deep nasal) swabs were collected according to national guidelines using dry FLOQSwabs^®^ (Copan, Brescia, Italy). Briefly, FLOQSwabs were eluted in 500 μL of phosphate buffered saline (PBS) and 200 μL of eluent was used for nucleic acid extraction using the Roche Magnapure 96 extraction system (Roche, Basel, Switzerland), according to the manufacturer’s instructions. The majority of SARS-CoV-2 samples were initially tested using the LightMix^®^ Modular SARS and Wuhan CoV E-gene kit (targeting the E-gene; sensitivity 96.5%, specificity of 98.5% (13); TIB Molbiol, Berlin, Germany) using 10 μL nucleic acid extract, according to the manufacturer’s instructions. RT-PCR was performed on the LightCycler 480 II Real-Time PCR System (Roche). Some patient samples were also tested for SARS-CoV-2 using the AusDiagnostics Respiratory Pathogens 16-well assay (Mascot, Australia), on the AusDiagnostics High-Plex 24 system [the SARS-CoV-2 target of this assay is the ORF-1 gene; 98.4% positive agreement with the Victorian Infectious Diseases Reference Laboratory (VIDRL) reference assay (14)]. The assay also detects other respiratory pathogens including influenza A/B, RSV A/B, rhinovirus, enterovirus, parechovirus, adenovirus, human parainfluenza viruses 1-4, human metapneumovirus, *Bordetella pertussis* and *Mycoplasma pneumoniae*), using 10 μL nucleic acid extract. Except in a small number of cases where there was insufficient sample, samples that were SARS-CoV-2-positive on a screening assay with a single gene target were confirmed by testing on a second assay. C_t_ values at diagnosis for SARS-CoV-2-positive patients are provided, when available, in the extended data.

### Flow Cytometry of PBMC Samples

Blood was collected in EDTA tubes from each participant and processed into peripheral blood mononuclear cells (PBMC) (15). For flow cytometry analysis of freshly isolated PBMC, cells were washed in 1 mL PBS prior to viability staining using BV510 viability dye according to manufacturer’s instructions. The viability dye reaction was stopped by the addition of FACS buffer (2% heat-inactivated FCS in 2 mM EDTA) and cells were centrifuged at 350 x g for 5 minutes. Cells were then resuspended in human FC-block according to manufacturer’s instructions for 5 minutes at room temperature. The antibody cocktail (**Supplementary Table 1**) made up at 2X concentration was added 1:1 with the cells and incubated for 30 minutes on ice. Following staining, cells were washed with 2 mL FACS buffer and centrifuged at 350 x g for 5 minutes. Cells were then resuspended in 2% PFA for a 20-minute fixation on ice, washed, and resuspended in 150 µl FACS buffer for acquisition using the BD LSR X-20 Fortessa (BD Biosciences, New Jersey, United States). For all flow cytometry experiments, compensation was done at the time of sample acquisition using compensation beads. **Supplementary Figure 1** depicts the manual gating strategy for all samples.

### Data Analysis

Flow cytometry data were analysed using FlowJo Version 10.7.1 software. Uniform Manifold Approximation and Projection (UMAP) analyses were conducted using concatenated files containing 5,000 randomly selected live single cells per sample. Manually gated results are presented as proportion of live cells. For CD4 and CD8 T cell subset analyses, results are presented as proportion of parent gate. Data was plotted in Prism version 8.0.0.

**Figure 1:**
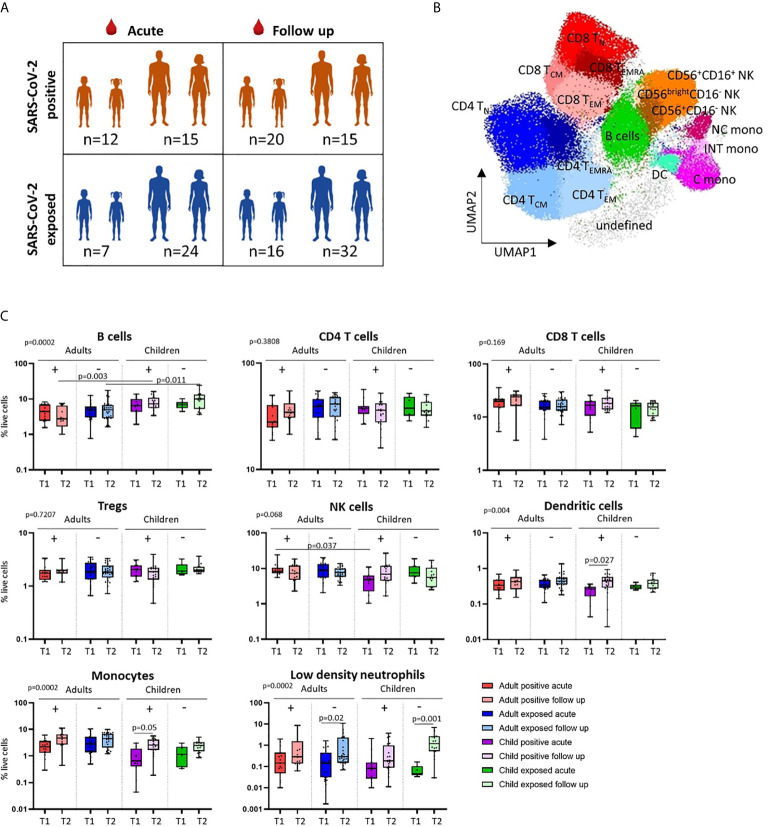
Cross-sectional analysis of immune cell profiles in SARS-CoV-2 positive and SARS-CoV-2 exposed children and adults. **(A)** Blood samples were collected during infection/exposure (acute) or 4-9 weeks following (follow up) from SARS-CoV-2 positive participants (n=12 child acute, n=20 child follow up, n=15 adult acute, n=15 adult follow up) and SARS-CoV-2 exposed participants (n=7 child acute, n=16 child follow up, n=24 adult acute, n=32 adult follow up). **(B)** Blood samples were processed into PBMC and analysed on the day of collection by flow cytometry. Unsupervised analysis of flow cytometry data revealed 17 clusters associated with CD4 T cells, CD8 T cells, NK cells, monocytes, dendritic cells, B cells and their cell subsets. **(C)** Comparison of the proportions of major cell populations in each clinical group (T1 – acute samples, T2 – follow up samples). P values by Kruskal-Wallis rank sum test and Dunn’s multiple comparison testing. FDR-adjusted P-values are reported. Boxplots show the medians, the 1^st^ and 3^rd^ quartile as well as the smallest and largest values as whiskers.

**Figure 2:**
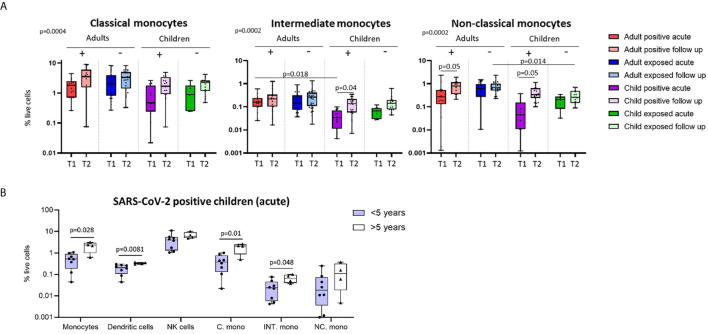
Innate cell responses in children and adults. **(A)** Classical, intermediate and non-classical monocyte proportions from SARS-CoV-2 positive participants (n=12 child acute, n=20 child follow up, n=15 adult acute, n=15 adult follow up) and SARS-CoV-2 exposed participants (n=7 child acute, n=16 child follow up, n=24 adult acute, n=32 adult follow up), T1 – acute samples, T2 – follow up samples. **(B)** Innate cell profiles in SARS-CoV-2 positive children during infection, stratified by age [under 5 years (n=8, median age 1.5 years), over 5 years (n=4, median age 10.5 years)]. P-values by Kruskal-Wallis rank sum test and Dunn’s multiple comparison testing **(A)** and by Mann-Whitney U test **(B)**. FDR-adjusted P-values are reported. Boxplots show the medians, the 1^st^ and 3^rd^ quartile as well as the smallest and largest values as whiskers.

**Figure 3:**
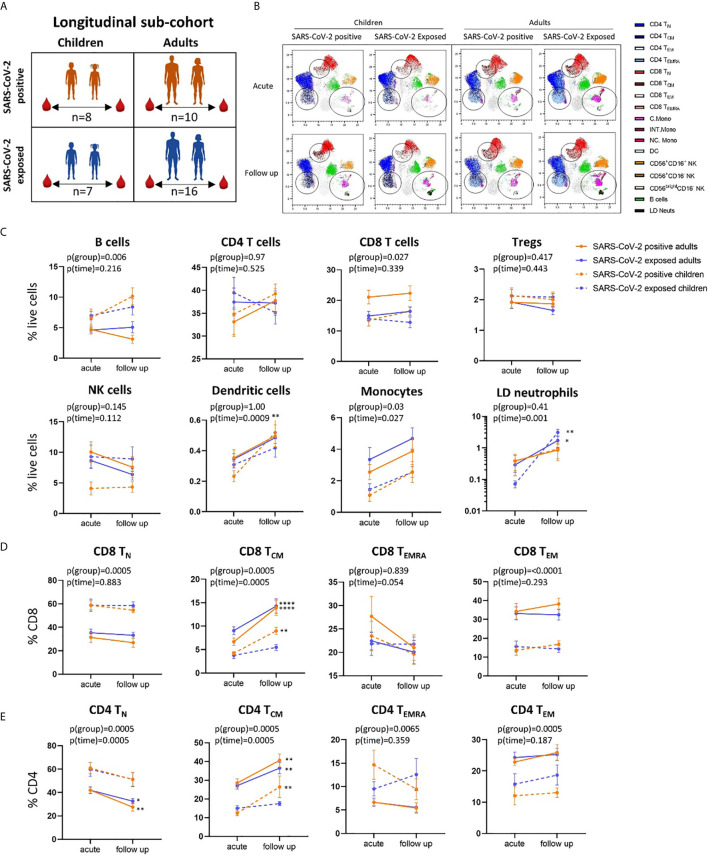
Longitudinal analysis of immune cell profiles in SARS-CoV-2 positive and SARS-CoV-2 exposed children and adults. **(A)** Paired blood samples were collected during infection/exposure and 4-9 weeks following from n=8 SARS-CoV-2 positive children, n=10 SARS-CoV-2 positive adults, n=7 SARS-CoV-2 exposed children and n=16 SARS-CoV-2 exposed adults. **(B)** UMAP plots consisting of cells from each individual from each clinical group. Circles highlight changes in frequency over time in key cell clusters: monocytes, low density neutrophils, CD8 T_CM_ and CD4 T_CM_ subsets. **(C)** Longitudinal analysis of major immune cell proportions (by manual gating) in each clinical group. **(D)** CD8 and **(E)** CD4 T cell subsets in each clinical group. Naive T cells (T_N_), central memory T cells (T_CM_), effector T cells (T_EMRA_) and effector memory T cells (T_EM_). P-values by two-way repeated measures analysis of variance with Sidak’s multiple comparison testing. FDR-adjusted P-values are reported, *p < 0.05, **p < 0.01, ****p < 0.0001. For **(C–E)**, mean ± SEM are shown.

**Figure 4:**
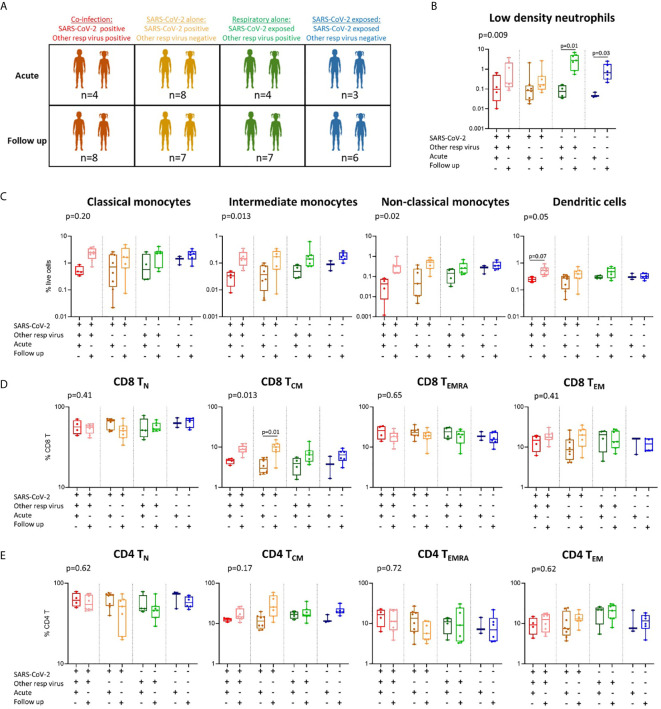
Immune profiles in children with SARS-CoV-2 single infection, SARS-CoV-2 co-infection, and non-SARS-CoV-2 respiratory infection. **(A)** A stratified analysis of samples collected from children with SARS-CoV-2 co-infection [SARS-CoV-2 positive, other respiratory virus positive (n=4 acute, n=8 follow up)], SARS-CoV-2 infection alone [SARS-CoV-2 positive, other respiratory virus negative (n=8 acute, n=7 follow up)], respiratory virus alone (SARS-CoV-2 negative (exposed), other respiratory virus positive (n=4 acute, n=7 follow up)], and SARS-CoV-2 exposed alone (SARS-CoV-2 negative, other respiratory virus negative (n=3 acute, n=6 follow up)]. **(B)** Proportions of low-density neutrophils. **(C)** Proportions of monocyte subsets and dendritic cells. **(D)** CD8 T cell naïve (T_N_), central memory (T_CM_), effector (T_EMRA_) and effector memory (T_EM_) populations in each group. **(E)** CD4 T cell naïve (T_N_), central memory (T_CM_), effector (T_EMRA_) and effector memory (T_EM_) populations in each group. P values by Kruskal-Wallis rank sum test and Dunn’s multiple comparison testing. FDR-adjusted P-values are reported. Boxplots show the medians, the 1^st^ and 3^rd^ quartile as well as the smallest and largest values as whiskers.

For differential abundance analysis of all groups in cross-sectional cohorts (**Figures 1**, **2**, **4**), p-values were determined by Kruskal-Wallis rank sum test and adjusted for multiple comparisons (x23 cell populations) using the Benjamini and Hochberg approach to control the false discovery rate (FDR) (16). FDR-adjusted p<0.05 were considered significant. For cell types showing an FDR-adjusted significant difference, Dunn’s multiple comparison testing was used to find differences between clinical groups. For statistical testing of the longitudinal cohort (**Figure 3**), p-values were determined by two-way repeated measures analysis of variance and adjusted for multiple comparisons [x46 (23 cell populations, p(group) and p(time))] using the Benjamini and Hochberg approach to control the false discovery rate (FDR). FDR-adjusted p<0.05 were considered significant. For cell types showing an FDR-adjusted significant difference, Sidak’s multiple comparison testing was used to find differences over time. All statistical analysis was performed in Prism version 9.0.0, with multiple comparison correction performed in RStudio version 4.0.3. Boxplots show the medians, the 1^st^ and 3^rd^ quartiles as well as the smallest and largest values as whiskers. Individual data points are shown.

## Results And Discussion

### Comparison of Immune Cell Profiles in Children and Adults

Clinical characteristics and demographics of the patients in this study are shown in **Table 1**. We first investigated differences in the circulating immune cell profile between children and adults using high dimensional flow cytometry and analysis on freshly isolated PBMCs. Children were aged between 1 and 17 years, and adults between 19 and 62 years (**Figure 1A**). UMAP analysis of all samples revealed clusters associated with CD4 T cells [naïve (T_N_), central memory (T_CM_), effector CD45RA^+^ (T_EMRA_) and effector memory (T_EM_)], CD8 T cells [naïve (T_N_), central memory (T_CM_), effector CD45RA^+^ (T_EMRA_) and effector memory (T_EM_)], B cells, NK cells (CD56^+^CD16^+^, CD56^+^CD16^-^ and CD56^bright^CD16^-^), monocytes (classical: CD14^+^CD16^-^, intermediate: CD14^+^CD16^+^, non-classical: CD14^low^CD16^+^) and dendritic cells (**Figure 1B**). Children had significantly higher proportions of CD4 T_N_ and CD8 T_N_ cells, whilst adults showed a clear shift toward CD4 and CD8 T memory cell populations (**Supplementary Figure 2A**). Children also had higher proportions of circulating B cells relative to adults (**Figure 1C** and **Supplementary Figure 2A**). While studies investigating the complete immune cell profile of children are rare, our findings are consistent with previous reports of cell subsets investigated in isolation that show a shift from naive to memory T cells with age, as well as higher proportions of circulating B cells in children relative to adults (17, 18).

### SARS-CoV-2-Positive Children Show Marked Alterations in Myeloid Cells During Infection

We next explored cross-sectional differences in immune cell profiles between children and adults with or exposed to SARS-CoV-2. Samples were collected during or approximately 4-to-9 weeks post infection/exposure from SARS-CoV-2-positive and SARS-CoV-2-exposed children and adults. No significant differences in proportions of total CD4 T cells, total CD8 T cells or regulatory T cells (Tregs) were observed between children and adults with or exposed to SARS-CoV-2 (**Figure 1C**).

We found that SARS-CoV-2-positive children had lower proportions of dendritic cells and monocytes during the acute phase relative to follow up [median proportion in SARS-CoV-2 positive children acute *vs* follow up – dendritic cells: 0.23% *vs* 0.44% (p=0.02), and monocytes: 1.04% *vs* 2.75% (p=0.05)] (**Figure 1C**). Further analysis of monocyte subsets revealed lower proportions of both the intermediate and non-classical monocyte subsets in SARS-CoV-2-positive children during infection compared to the same group at follow up [median proportion acute *vs* follow up – intermediate monocytes: 0.04% *vs* 0.15% (p=0.04), and non-classical monocytes: 0.09% *vs* 0.44% (p=0.05)] (**Figure 2A**). SARS-CoV-2-positive adults also had reduced proportions of non-classical monocytes during the acute phase compared to follow up [median proportion of non-classical monocytes in SARS-CoV-2 positive adults acute *vs* follow up: 0.44% *vs* 0.91% (p=0.05)] (**Figure 2A**). This is consistent with our previous findings in an interim analysis of this cohort, revealing low circulating proportions of monocyte subsets and dendritic cells in SARS-CoV-2-positive children (19). Previous work by our team using pre-pandemic PBMCs from healthy children aged 1-15 years shows an average circulating frequency of 0.8% for dendritic cells and 4.8% for monocytes (20, 21), further highlighting the reduced proportions observed during the acute phase in SARS-CoV-2 positive children.

It should be noted that our sampling strategy for the complete cohort involved a mix of repeated sampling from the same participants as well as single sampling from participants at either timepoint, whichever was feasible. This may lead to some selection bias in the analysis for those with repeated sampling. However, we conducted a sensitivity analysis of only participants with single samples, revealing the same changes over time in these cell populations (see Extended Data).

Several other reports have also shown reduced proportions of monocytes and NK cells in the blood of adults with COVID-19, possibly reflecting redistribution of these cells to the lung (22–25). Single cell sequencing studies of bronchoalveolar lavage (BAL) samples from patients with varying COVID-19 severity provide further evidence for infiltration of innate cells into the lung during acute infection (26). Alveolar macrophages and dendritic cells have been shown to be enriched in the BAL of patients with mild COVID-19 disease, whilst severe disease is associated with infiltration of neutrophils, NK cells and monocyte-derived macrophages (27). A recent single cell analysis of lung tissue from adults with lethal COVID-19 revealed dense infiltration of monocyte-derived macrophages, impaired T cell responses, as well as monocyte and epithelial cell-derived inflammatory cytokines (28).

### Immune Responses in Younger and Older Children During Acute Infection

Combined, our results suggest that SARS-CoV-2-positive children show more pronounced changes in innate immune cell populations compared to infected adults. These results may, in part, provide a mechanistic explanation for the reduced susceptibility and severity of SARS-CoV-2 infection in children compared to adults (1). Recent data suggests further reduced severity and transmission of SARS-CoV-2 in younger children compared to adolescents (2, 4, 29). We therefore compared immune cell profiles in SARS-CoV-2-positive children under five years of age (median age 1.5 years) to those over age five (median age 10.5 years) during the acute phase of infection (**Figure 2B**). SARS-CoV-2-positive children under five showed significantly lower proportions of circulating monocytes and dendritic cells during infection compared to SARS-CoV-2-positive children over the age of five [median proportion during infection in children under five *vs* over five – monocytes: 0.5% *vs* 2.26% (p=0.02), and dendritic cells: 0.2% *vs* 0.32% (p=0.008)]. Within the monocyte subpopulations, significantly lower proportions of both classical and intermediate monocytes were observed in SARS-CoV-2-positive children under five relative to positive children over the age of five [median proportion during infection in children under five *vs* over five – classical monocytes: 0.38% *vs* 2.07% (p=0.01) and intermediate monocytes: 0.02% *vs* 0.06% (p=0.04)] (**Figure 2B**). Innate immune differences between infected children and infected adults were therefore most evident in infants and pre-school aged children.

Few studies have directly compared immune responses to SARS-CoV-2 infection in children and adults. One recent study compared cytokine, humoral and cellular immune responses in children and adults hospitalised with COVID-19. This revealed that children had a shorter length of hospital stay, decreased requirement for ventilation and lower mortality compared to adults. SARS-CoV-2 specific CD4 T cell responses and neutralising antibody titres were higher in adults compared to children (30).

A comparison of severe paediatric COVID-19, MIS-C and severe adult COVID-19 revealed that MIS-C patients had similar levels of T cell activation as severely ill adults. Robust activation of CX3CR1^+^ CD8 T cells was unique to MIS-C patients, whose immune activation resolved with treatment and recovery (31). Systems serology analysis of antibodies in children and the elderly revealed that whilst elderly individuals induce more class switched antibodies that target cross-reactive regions of SARS-CoV-2, infected children have antibodies with enhanced Fc functions that are more effective in clearing the virus (32).

### Temporal Changes in CD8 and CD4 T Cell Central Memory Subsets in SARS-CoV-2-Positive Children and Adults

Using longitudinally collected samples from the same patients during infection and up to 9 weeks post-infection (**Figures 3A, B** and **Supplementary Figure 3**), we show a significant increase in the proportion of dendritic cells and monocytes over time (p=0.0009 and p=0.02, respectively) (**Figure 3C**). Post-hoc testing revealed that SARS-CoV-2-positive children showed a significant increase in dendritic cells over time (median proportion during infection 0.23% *vs* follow up 0.52%, p=0.008). Changes in cell frequency over time in children and adults are highlighted by the UMAP analyses in **Figure 3B**.

Children and adults had significant differences in the proportion of naïve and effector CD8 and CD4 T cell populations at both time points that were independent of SARS-CoV-2 status and related to age (**Figures 3D, E** and **Supplementary Figure 2C, D**). However, central memory CD8 T cells (CD8 T_CM_) significantly increased over time in SARS-CoV-2-infected children and adults [median proportion of CD8 T_CM_ acute *vs* follow up – SARS-CoV-2 positive children: 4.22% *vs* 8.99% (p=0.006), SARS-CoV-2 positive adults: 6.67 *vs* 13.91 (p<0.0001)] (**Figure 3D**). Central memory CD4 T cells (CD4 T_CM_) also showed the same response, significantly increasing over time in SARS-CoV-2 infected children and adults [median proportion of CD4 T_CM_ acute *vs* follow up – SARS-CoV-2 positive children: 12.4% *vs* 26.41% (p=0.001), SARS-CoV-2 positive adults: 28.5% *vs* 40.59% (p=0.002)] (**Figure 3E**). We also report significant decreases in proportion of naïve CD4 T cells (CD4 T_N_) in SARS-CoV-2-positive adults between the acute and follow up phases [median proportion 41.97% *vs* 27.53% (p=0.001)].

These results highlight that both CD8 and CD4 central memory T cells may play key roles in the immune response to SARS-CoV-2 in both children and adults, with increases in this cell population observed during the convalescent period in participants diagnosed with COVID-19. Previous work by our team using pre-pandemic PBMCs from healthy children (11-14 years) shows an average frequency of 5.12% for CD8 T_CM_, further highlighting the increase in CD8 T_CM_ in the SARS-CoV-2 positive children (20). A study analysing longitudinal samples from adults with mild COVID-19 showed the presence of SARS-CoV-2 specific memory CD8 and CD4 T cells out to 6 months post infection (33). SARS-CoV-2 specific CD8 T cells consisted primarily of T_EMRA_ phenotype with small populations of T_CM_ and T_EM_ cells. SARS-CoV-2 specific CD4 T cells, however, were primarily of T_CM_ or T_EM_ phenotype (33). SARS-CoV-2 specific CD8 and CD4 T cells were also identified at 8 months post infection in another study comparing mild and severe disease, with greater memory CD4 T cell cytokine responses observed in severe patients relative to those of asymptomatic patients (34). Similarly, significantly larger overall T cell responses were observed in convalescent patients who had severe compared with mild disease, however mild disease was associated with greater SARS-CoV-2 specific CD8 T cell responses (35).

### SARS-CoV-2-Exposed Children and Adults Show Immune Cell Changes Over Time

SARS-CoV-2-exposed but PCR-negative close contact participants also had changes in their immune cell profiles over time, despite no evidence of confirmed infection. Exposed PCR-negative children and adults both had increases in low-density immature neutrophils (defined as SSC^high^CD16^+^ cells in PBMCs) at follow up compared to acute phase [median proportion of low-density neutrophils acute *vs* follow up – exposed children: 0.07% *vs* 1.95% (p=0.001), exposed adults: 0.31% *vs* 1.42% (p=0.02)] (**Figure 1C** and **Figure 3B**). These neutrophils, with reduced granularity compared to conventional neutrophils and observed in mononuclear low density cell fractions, have been reported in adults with severe COVID-19 and may represent emergency myelopoiesis (36–38). CD8 T_CM_ and CD4 T_CM_ also increased over time in SARS-CoV-2-exposed adults [median proportion acute *vs* follow up – CD8 T_CM_: 9.04% *vs* 14.28% (p<0.0001), CD4 T_CM_: 27.10 *vs* 36.45 (p=0.0034)], but not in SARS-CoV-2-exposed children [median proportion acute *vs* follow up - CD8 T_CM_: 3.8% *vs* 5.46% (p=0.70), CD4 T_CM_: 15% *vs* 17.5% (p=0.94)] (**Figures 3D, E**).

As the changes in the SARS-CoV-2-exposed group over time were not antigen-specific, we next explored whether these responses were influenced by infection with other respiratory viruses in some of our SARS-CoV-2-exposed participants. We compared SARS-CoV-2 participants with or without infection with other respiratory viruses at both acute and follow up timepoints. This showed that the changes observed in key cell types over time in the exposed groups – low density neutrophils in both children (**Figure 4B**) and adults (**Supplementary Figure 4**), as well as CD8 T_CM_ and CD4 T_CM_ in adults (**Supplementary Figure 4**) – occurred in all exposed participants, independent of the presence of other respiratory viruses. In fact, changes over time in CD8 T_CM_ and CD4 T_CM_ cells were most evident in SARS-CoV-2-exposed adults without co-infection with other respiratory pathogens [median proportion acute *vs* follow up - CD8 T_CM_: 9.14% *vs* 16.00% (p=0.0034), CD4 T_CM_: 27.89% *vs* 42.54% (p=0.0001)] (**Supplementary Figure 4**).

As close contact exposed participants in our cohort were all repeatedly negative upon SARS-CoV-2 PCR testing, these results suggest that exposure in the household can lead to the generation of T cell immunity even in the absence of confirmed infection. These results may also suggest that the exposed participants were infected with a low viral load that was undetectable by PCR testing. This observation is supported by a recent study exploring memory T cell responses in household close contacts (exposed but PCR negative) of confirmed COVID-19 patients, showing that both COVID-19 patients and their close contacts generate SARS-CoV-2 specific CD8 and CD4 T cell memory responses (39). The authors hypothesised that the generation of virus specific T cell immunity in SARS-CoV-2-exposed individuals in the absence of infection was either due to exposure to a limited number of viral particles or exposure for short time. In a previous case study of two parents with PCR-confirmed symptomatic SARS-CoV-2 infection and their three SARS-CoV-2 PCR negative children, we also showed that exposed children can mount a cellular immune response characterised by changes in both innate and adaptive immune cells (40).

### Differences in Immune Responses Between Children With SARS-CoV-2 Infection Alone, SARS-CoV-2 Co-Infection, and Other Respiratory Viral Infections

As we were interested to explore differences in immune responses between children with COVID-19 and other respiratory infections, we compared children with SARS-CoV-2 single infection (SARS-CoV-2-positive, respiratory-negative), SARS-CoV-2 co-infection (SARS-CoV-2-positive, respiratory-positive) and children with non-SARS-CoV-2 respiratory viral infection (SARS-CoV-2 negative, respiratory-positive) (**Figure 4A** and **Supplementary Figure 5**). These results show that the SARS-CoV-2 single- and co-infected children show the greatest change in response over time in monocyte subpopulations and dendritic cells (**Figure 4C**), as well as CD8 T_CM_ cells (**Figure 4D**), relative to non-SARS-CoV-2 respiratory-positive children. Children with SARS-CoV-2 co-infection show the lowest proportions of intermediate and non-classical monocytes at the acute timepoint (intermediate: 0.03% and non-classical: 0.04%) followed by SARS-CoV-2 single infected children (intermediate: 0.04% and non-classical: 0.12%), non-SARS-CoV-2 respiratory infected children (intermediate: 0.05% and non-classical: 0.14%), and finally SARS-CoV-2 negative respiratory-negative children (intermediate: 0.08% and non-classical: 0.25%). The lowest proportion of circulating dendritic cells was also observed in the SARS-CoV-2 co-infected children during the acute phase (**Figure 4C**). SARS-CoV-2 single-infected children were the only group to show a significant increase in CD8 T_CM_ cells over time (median 3.8% acute *vs* 9.7% follow up), however this trend was also observed in SARS-CoV-2 co-infected children over time (median 4.5% acute *vs* 8.8% follow up) and non-SARS-CoV-2 respiratory infected children (3.7% acute *vs* 6.9% follow up) (**Figure 4D**). Similar results were also seen for CD4 T_CM_ cells in SARS-CoV-2 single-infected children (median 11.8% acute *vs* 31.7% follow up) and co-infected children (median 12.4% acute *vs* 16.9% follow up) but not in non-SARS-CoV-2 respiratory infected children (median 16.5% acute *vs* 18.0% follow up) (**Figure 4E**). Combined, these data suggest that SARS-CoV-2 induces a greater change in monocyte, dendritic cell, CD8 T_CM_ and CD4 T_CM_ responses in children compared to other common childhood respiratory viruses. However, we acknowledge the small numbers in our groups and these results should be confirmed in larger studies.

## Conclusion

In summary, we characterised the circulating immune cell landscape of children and adults with mild COVID-19, as well as their PCR-negative close contacts during and up to 9 weeks post infection. We directly compared immune cell profiles in children and adults, revealing markedly low proportions of circulating innate immune cells in SARS-CoV-2-positive children during acute infection, most evident in children under the age of five. These results suggest enhanced recruitment of these circulating innate immune cells to sites of infection, such as the lung. The generation of CD8 T_CM_ and CD4 T_CM_ cells up to 9 weeks post infection were common to both SARS-CoV-2-positive children and adults. Exposure to SARS-CoV-2 in household close contacts also resulted in a change in immune cells over time despite no evidence of confirmed SARS-CoV-2 infection and independent of the presence of other respiratory viruses. This response was characterised by increases in low-density neutrophils at follow up, as well as increases in CD8 T_CM_ and CD4 T_CM_ over time in exposed adults. A comparison of immune responses in children with COVID-19 to children with other common respiratory infections revealed that SARS-CoV-2 infection induced a greater change in innate and T cell mediated immune responses. Our work adds to recent studies exploring immunity and clinical outcomes in children with mild COVID-19 (19, 32, 41) and provides new mechanistic insights into the immune response during and after recovery from COVID-19 in both children and adults.

## Data Availability Statement

The original contributions presented in the study are included in the article/**Supplementary Material**. Further inquiries can be directed to the corresponding author.

## Ethics Statement

This project was approved by The Royal Children’s Hospital Melbourne Human Research Ethics Committee (HREC): HREC/ 63666/RCHM-2019. All participants or their legal guardians provided written informed consent.

## Author Contributions

MN, AS, DB, RS, ST, and NWC designed the study. ST, KD, and VC collected the clinical data and specimens. MN, SB, and VC performed the experiments. MN, RS, ST, and NWC interpreted the findings and wrote the original manuscript. IO, JN, ZT, JA, CD, SS, LD, CM, PL, VI, PM, JB, KM, NC, and SM are study investigators and assisted in interpreting the findings. All authors contributed to the article and approved the submitted version.

## Funding

The FFX study has Australian Commonwealth government support for identification of positive samples and database management. Sample analysis was supported by the Royal Children’s Hospital Foundation.

## Conflict of Interest

The authors declare that the research was conducted in the absence of any commercial or financial relationships that could be construed as a potential conflict of interest.

## Publisher’s Note

All claims expressed in this article are solely those of the authors and do not necessarily represent those of their affiliated organizations, or those of the publisher, the editors and the reviewers. Any product that may be evaluated in this article, or claim that may be made by its manufacturer, is not guaranteed or endorsed by the publisher.
