# Supplementary material for: Children and Adults in a Household Cohort Study Have Robust Longitudinal Immune Responses Following SARS-CoV-2 Infection or Exposure
Source: Front Immunol. 2021 Oct 13;12:741639. doi: 10.3389/fimmu.2021.741639 (PMC8548628; doi:10.3389/fimmu.2021.741639)
Supplement: Supplementary file 1 [file DataSheet_1.pdf]

SUPPLEMENTAL FIGURES AND TABLE

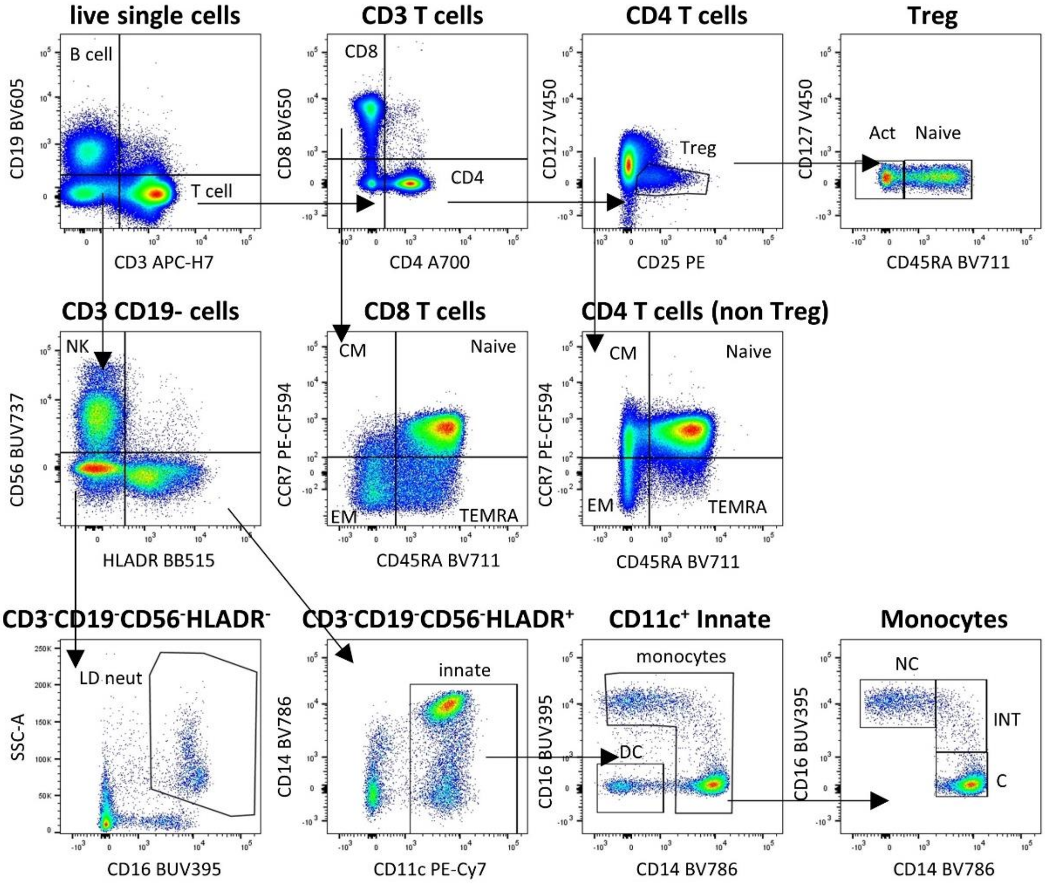

Supplementary Figure 1. Representative flow cytometry gating strategy for PBMC samples. Within the live single cell fraction, B cells were selected based on CD19 expression, and the total T cell fraction based on CD3 expression. CD4 and CD8 T cells, and their naïve, effector, memory and regulatory (Treg) subsets were also quantified. $CD3^-CD19^-$ cells were classified into NK cells ($CD56^+$) and innate cells ($HLA\text{-}DR^+$). Writhin the innate cell fraction, $CD14^+$ monocytes and $CD11c^+$ DCs were identified. Monocyte subsets were identified based on CD16 expression and classified into classical, intermediate, and non-classical subsets. Low density neutrophils were observed in the PBMC fraction at convalescence, characterised by a high SSC profile and CD16 expression.

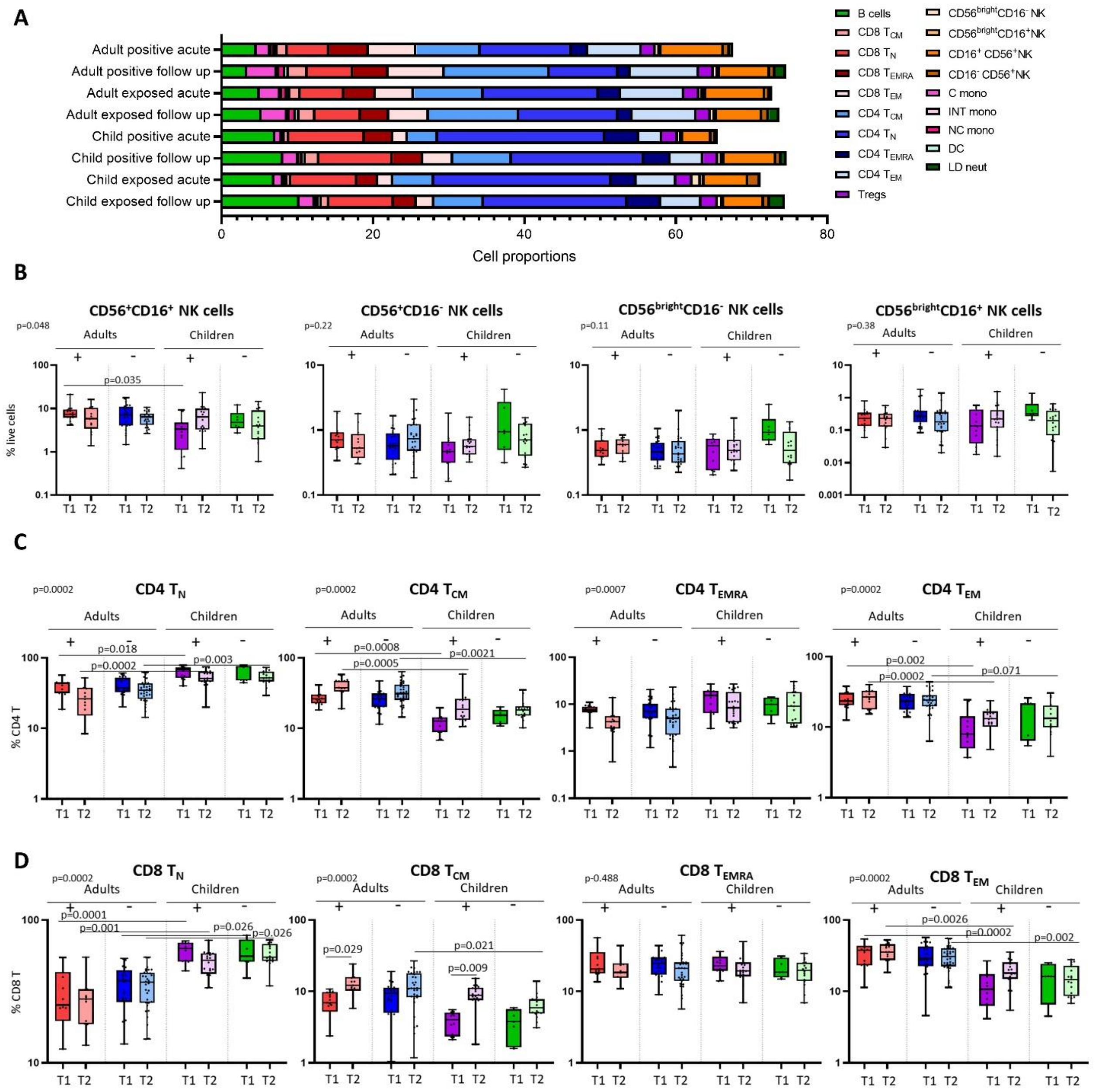

Supplementary Figure 2. Proportions of monocyte, NK cell and CD4 T cell subsets in the cross-sectional complete cohort. **(A)** Mean proportions of all immune cell populations in each clinical group. **(B)** Proportions of $CD56^+CD16^+$, $CD56^+CD16^-$. $CD56^{bright}CD16^-$ and $CD56^{bright}CD16^+$ NK cells in each clinical group. Proportions of **(C)** CD4 T and **(D)** CD8 T cell naïve, central memory, effector and effector memory populations in each clinical group. P values by Kruskal-Wallis rank sum test and Dunn's multiple comparison testing. FDR-adjusted P-values are reported. Boxplots show the medians, the 1st and 3rd quartile as well as the smallest and largest values as whiskers.

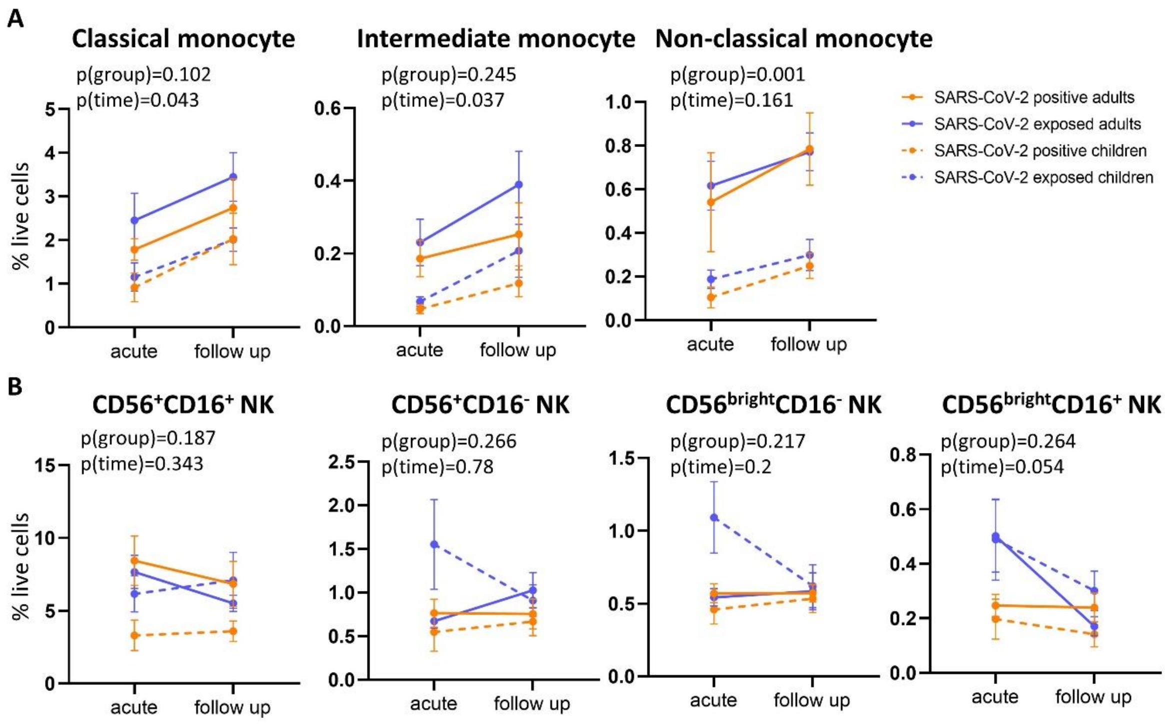

Supplementary Figure 3. Proportions of monocyte and NK cell subsets in the longitudinal sub-cohort. **(A)** Proportions of classical ($CD14^+CD16^-$), intermediate ($CD14^+CD16^+$) and non-classical ($CD14^{low}CD16^+$) monocytes in each clinical group. **(B)** Proportions of $CD56^+CD16^+$, $CD56^+CD16^-$. $CD56^{bright}CD16^-$ and $CD56^{bright}CD16^+$ NK cells in each clinical group. P-values by two-way repeated measures analysis of variance with Sidak's multiple comparison testing. FDR-adjusted P-values are reported and mean ± SEM are shown.

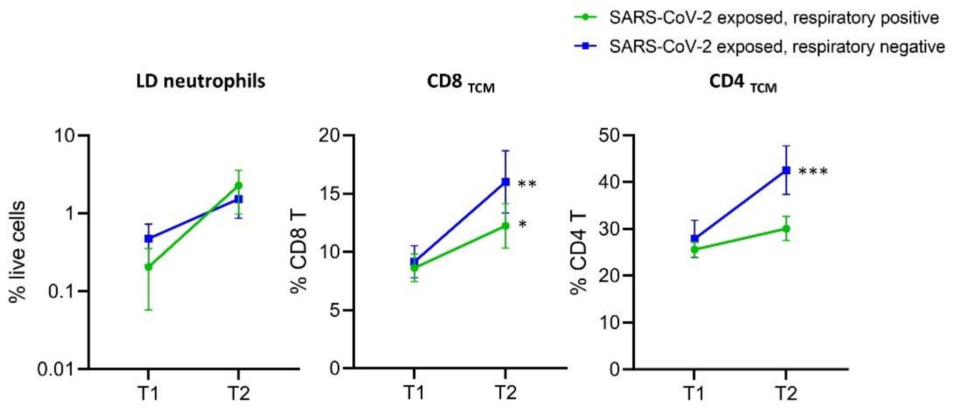

Supplementary Figure 4. Cell populations of interest in SARS-CoV-2 exposed adults. Matched samples collected from SARS-Cov-2 exposed adults with other respiratory viruses (n=8, green) and SARS-CoV-2 exposed adults without detection of other respiratory viruses (n=7, blue). T1: acute, T2: follow up. P-values by two-way repeated measures analysis of variance with Sidak's multiple comparison testing, *p<0.05, **p<0.01, ***p<0.001. Mean ± SEM are shown.

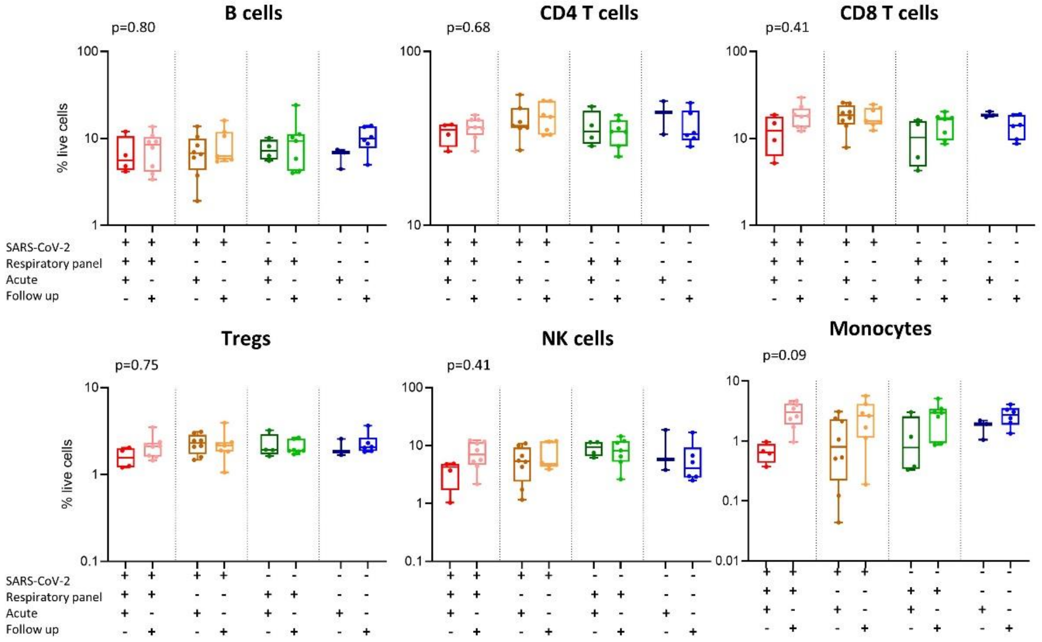

Supplementary Figure 5. Main cell types in children with SARS-CoV-2 infection alone, SARS-CoV-2 co-infection, and non-COVID-19 respiratory infection. T1: acute, T2: follow up. P values by Kruskal-Wallis rank sum test and Dunn's multiple comparison testing. FDR-adjusted P-values are reported. Boxplots show the medians, the 1st and 3rd quartile as well as the smallest and largest values as whiskers.

Supplementary Table 1. Flow cytometry cocktail

Surface Marker	Fluorophore	Clone	Final Dilution
CD25	PE	M-A251	1:25
CD127	V450	HIL7RM21	1:50
CD3	APCH7	SK7	1:50
CD14	BV786	M5E2	1:50
CD45RA	BV711	HI100	1:100
HLADR	BB515	G46-6	1:100
CD56	BUV737	NCAM16.2	1:100
CD11c	PE-Cy7	B-ly6	1:100
CD4	A700	RPA-T4	1:100
CCR7	PE-CF594	150503	1:200
CD19	BV605	SJ25C1	1:200
CD8	BV650	RPA-T8	1:200
CD16	BUV395	3G8	1:400
Live/dead	BV510		
